# Effects of Acetic Acid and Morphine in Shore Crabs, *Carcinus maenas:* Implications for the Possibility of Pain in Decapods

**DOI:** 10.3390/ani14111705

**Published:** 2024-06-06

**Authors:** Stuart Barr, Robert W. Elwood

**Affiliations:** School of Biological Sciences, Queen’s University, Belfast BT9 5DL, UK; s_barr@hotmail.com

**Keywords:** autotomy, pain, acetic acid, morphine, *Carcinus maenas*, decapod

## Abstract

**Simple Summary:**

Injection of acetic acid into one leg of a crab caused rubbing of that leg and holding the leg off the floor of a tank. Such activities directed towards the site of a noxious stimulus are consistent with the idea of pain. Some crabs also cast off the leg injected with acid. Because that occurred in association with possible pain-related behaviour, it too might be caused by pain. Prior morphine injection caused various behavioural changes, but morphine did not ameliorate the responses to acetic acid. Therefore, morphine does not act as an analgesic, and this agrees with previous work. Nevertheless, the directed behaviour that follows injection into a leg agrees with other studies and provides additional evidence suggesting that these animals experience pain.

**Abstract:**

Noxious chemicals, coupled with morphine treatment, are often used in studies on pain in vertebrates. Here we show that injection of morphine caused several behavioural changes in the crab, *Carcinus maenas*, including reduced pressing against the sides of the enclosure and more rubbing and picking at the mouth parts and, at least for a short time, more defensive displays. Subsequent injection of acetic acid into one rear leg caused rubbing of the injected leg and the injected leg was held vertically off the ground. These activities directed at or involving the specific leg are consistent with previous observations of directed behaviour following noxious stimuli and are consistent with the idea that decapods experience pain. Further, acetic acid but not injection of water induced autotomy of the injected leg in these animals. Because autotomy is temporally associated with directed behaviour, it is possible that the autotomy is a pain-related response. Acetic acid is clearly a noxious substance when applied to decapods. However, morphine had no effect on the activities associated with acetic acid injection and thus there is no evidence for an analgesic effect. Further, the injection of acetic acid did not interfere with behavioural effects of morphine. The activities directed towards the site of injection are like those observed with injection, or with external application, of various noxious substances and the present study adds to a growing body of knowledge about possible pain in decapods.

## 1. Introduction

Studies on mammalian pain frequently employ noxious chemicals, including acetic acid and formalin, to elicit marked behavioural changes that direct activities towards the site of injection, or other, more extreme, responses, such as writhing in mice, *Mus musculus*, that are interpreted as the animal being in pain [1,2]. The behaviour of these animals can be compared to others that are treated with potential analgesics to determine if those, presumably, pain responses are reduced [1,2].

This approach was taken in studies on fish to investigate if that taxon could experience pain [3]. The lips of trout were injected with either acetic acid or saline control, and the former rubbed their lips on the substrate and showed abnormal rocking, which was not seen in controls. However, morphine injection reduced the rubbing and rocking. This experiment provided ideas for an early study on potential pain experience in decapod crustaceans [4,5]. That study used glass prawns (*Palaemon elegans*), which had an antenna brushed with a local anaesthetic (benzocaine) or saline control and then later had the same antenna brushed with acetic acid or caustic soda or saline [5]. Without the local anaesthetic, the noxious chemicals caused a marked increase in rubbing the antenna against the walls of the tank, and grooming directed at the treated antenna, but these responses were reduced in those pretreated with benzocaine [5]. However, another study found no increase in behaviour directed to the site of application in three species of decapods following brushing of hydrochloric acid or caustic soda to an antenna [6]. That study concluded that decapods were not affected by chemicals of high or low pH. Several following studies questioned that conclusion. Shore crabs, *Carcinus maenas*, showed directed behaviour when their mouthparts or an eye was brushed with acetic acid [7], and glass prawns (*P. elegans*) used their chelipeds to rub an eye if the eye had been brushed with caustic soda [8]. Crabs (*Hemigrapsus sanguineus*) responded when formalin, another substance that causes pain in humans, was injected into a claw and the animal reduced the use of that appendage when walking and pressed that claw against its carapace [9]. Crabs also shook and rubbed the injected claw, and some autotomized the injected claw, but none of these activities were seen in saline-injected controls [9]. One aim of the present study is to ask if acetic acid, injected into a walking leg, induces behaviour that is directed at the injected leg. We also recorded autotomy, as that too might be mediated by pain [10]. This should answer the question of whether acetic acid can influence behaviour in a way that is consistent with the idea of pain in decapods. It also provides a novel method of assessing the effects of acetic acid because previous tests employed external application, whereas this is by injection.

While morphine has been shown to be a powerful analgesic in mammals, and reduces responses to various noxious stimuli, it may also have other, non-analgesic effects [11,12]. That is, morphine may cause changes in behaviour, and it is possible that these may mask, or be mistaken for, analgesia. This appeared to be the case when testing for analgesic effects of morphine in crustaceans. For example, responses to an electrical shock were reduced by dose-related morphine treatment in the mantis shrimp, *Squilla mantis* [13], and in the crab, *Chasmagnathus granulatus* [14]. In the latter, this apparent analgesia was reversed by the opioid antagonist, naloxone [14]. However, escape responses to a moving shadow *C. granulatus* were also reduced by morphine injection [15]. In this case, the behavioural effect of morphine cannot be attributed to analgesia. However, the effect could be due to a general reduction in perception or ability to move, and thus the proposed analgesic effect of morphine was questioned [15]. To test this idea, an experiment was devised in which crabs were repeatedly offered the choice to move to a dark shelter to escape a brightly lit area [16]. Some crabs were shocked within the shelter and some crabs had prior morphine treatment. The idea was that if morphine had an analgesic effect, then morphine-treated crabs would use the shelter even though they received a shock. It was found that crabs given morphine did not use the shelter in early trials, irrespective of being shocked or not, but did so in later trials. The conclusion was that morphine offered no analgesic effect for electric shock but did alter the general responsiveness or ability to move [16]. The present experiment further investigates the effects of morphine on the behavioural responses of shore crabs, *Carcinus maenas*, and tests for analgesic effects following acetic acid injection to a walking leg. It also tests for behaviour following acetic acid injection that is directed at the injected leg, which, if found, would be consistent with the idea of pain in crabs. In addition, it examines if autotomy occurs following acetic acid injection, as was noted with formalin [9], and if that reaction might be reduced by morphine.

## 2. Materials and Methods

### 2.1. Animal Collection and Care

European shore crabs (*Carcinus maenas*, carapace width = 5–8 cm) were collected from Bar Hall Bay, Strangford Lough (OS; J 617464), between May and July 2008, using baited pots. Only fully intact crabs were transported to Queen’s University, Belfast. They were housed in plastic tanks (76 × 38 × 17 cm) filled with aerated natural seawater, to a depth of 3 cm, and seaweed for shelter (*Ascophyllum nodosum*), in a cold room maintained at 11–13 °C with a twelve-hour light/dark regime. The crabs were fed fresh mackerel pieces every 2 days, after which the seawater was changed. Before performing the experiment, carapace width was measured and health status was noted once again as, during the storage time, some crabs were damaged due to conflicts. Only healthy, intact crabs were randomly assigned (by drawing tokens from a bag) to eight experimental groups (N = 12 per group), each of which involved two treatments and two observation periods in a 2 × 4 design. After use, the crabs were returned to the cold room and housed in new tanks, before being released back in Strangford Lough.

### 2.2. Experimental Treatments

#### 2.2.1. First Treatment

The first treatment was either a 100 µg/g injection of pharmaceutical-grade morphine sulphate solution (60 mg/2 mL), calculated by first weighing the animal to determine the correct volume, or an equivalent amount of distilled water. This dose was the same as that employed in previous studies [14,15,16]. This first injection was randomly chosen and administered in the cephalothoracic–abdominal membrane, using 0.3 mL insulin syringes, while the animal was briefly held on a treatment table. Following first treatment, the animal was placed into the observation tank (30 × 19 × 20 cm) containing fresh natural seawater (11–13 °C) to a depth of 1 cm. The observation tank was housed in an observation chamber (illuminated by a 60 W light bulb) behind a one-way mirror and behaviour recorded for 5 min.

Contact with the sides of the tank was recorded. This involved the crab pressing into the sides of the tank with its chelipeds, backing into the tank sides or in some cases attempting to climb the wall of the tank. The amount of time spent rubbing and picking the mouthparts with the chelipeds and the time spent in the lateral merus display (elevated carapace and extended chelipeds) was also recorded.

#### 2.2.2. Second Treatment

The crab was removed from the observation tank for the second treatment and again briefly held on the treatment table. The second injection was 0.2 mL of distilled water, 0.2 mL of 0.25% acetic acid, 0.2 mL of 0.5% acetic acid or 0.2 mL of 1.0% acetic acid. These concentrations of acetic acid were selected after preliminary trials. The treatment was randomly chosen and administered to the carpus/propus joint of a randomly chosen hind leg. This joint is about halfway along the length of the leg. Following treatment, the crab was placed into the observation tank again and behaviour recorded for 5 min. The same activities as before were recorded. In addition, rubbing of the injected leg with adjacent limbs, vertical extension of the injected limb, and incidence and timing of leg autotomy were recorded. Because morphine effects last for about 20 min [16], both observation periods were completed before the effects declined.

### 2.3. Statistical Methods

#### 2.3.1. First Observation Period

Effects of morphine vs. water injections on durations of contact with the sides of the tank, mouth rubbing and picking with the chelipeds, and defensive reaction were transformed to log(x + 1) and analysed using *t*-tests. The transformation improved the normal distribution of the data. The number of crabs showing defensive display responses was analysed using χ^2^ tests.

#### 2.3.2. Second Observation Period

The incidence of rubbing the injected leg, holding the injected leg vertically, and autotomy was analysed using χ^2^ tests for effect of first treatment (morphine or distilled water) and for effect of second treatment (distilled water or acetic acid). The durations of contact with the sides of the tank and of mouth rubbing and picking with the chelipeds were transformed to log(x + 1) and analysed using a 2-factor ANOVA (factor 1—morphine or distilled water; factor 2—distilled water, 0.25%, 0.5% and 1.0% acetic acid).

### 2.4. Ethical Considerations

This experiment was conducted in 2008, when there was little or no support for the idea of pain in decapod crustaceans. There were no legal restrictions on experiments and no requirement for ethical review on this group of animals within the United Kingdom. Nevertheless, we kept the numbers of animals low in each experimental group (*n* = 12 per group) and we kept the level of noxious stimulus as low as possible. All crabs recovered and were returned to the shore. Autotomy of a single leg does not impede locomotion and is not expected to interfere with feeding and mating. It thus poses less of a cost for the crab than does loss of a cheliped [17]. Further, the crabs were not fully grown, and autotomized walking legs should regenerate when the animal moults and grows. The results of similar experiments have changed the legal situation for decapods within the United Kingdom, which now recognizes that these animals are sentient. Despite this, there has been no change in the UK legal requirements for research on these animals and thus the experiment is fully compliant with current UK regulations. Nevertheless, we suggest that researchers should take the potential sentience of these animals into account when designing future studies rather than waiting for legal change and refer to guidelines on the use of wild animals [18] and, specifically, crustaceans [19].

## 3. Results

### 3.1. First Observation Period

**Figure 1:**
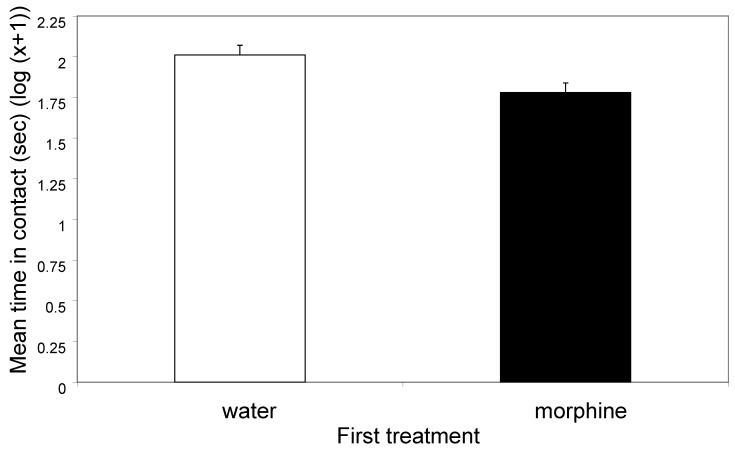
The mean (±SE) time (s) spent in contact with the sides of the tank in the first observation (log (x + 1)). The means equate to 100 s (water) and 60 s (morphine).

**Figure 2:**
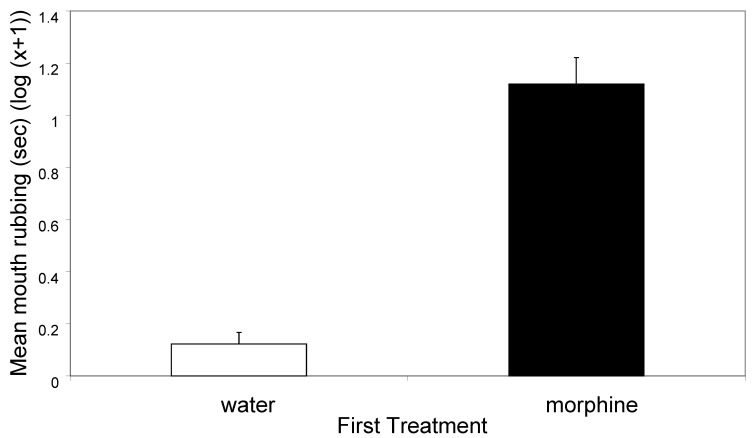
The mean (±SE) time (s) spent rubbing and picking the mouthparts with the chelipeds in the first observation (log(x + 1)). The means equate to 1.5 s (water) and 12.5 s (morphine).

Crabs that received morphine pressed against the sides of the tank for significantly less time (t_94_ = 2.92, *p* < 0.01; Figure 1), but rubbed and picked their mouthparts with their chelipeds significantly more (*t*_94_ = 8.96, *p* < 0.0001; Figure 2), and were more likely to show defensive displays than were those injected with water (20/48 for morphine and 2/48 for water, χ^2^_1_ = 17.04, *p* < 0.0001).

### 3.2. Second Observation Period

**Figure 3:**
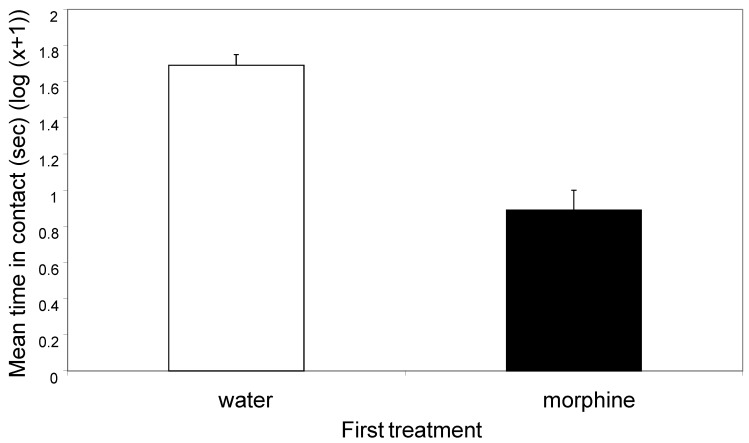
The mean (±SE) time (s) spent in contact with the sides of the tank in the second observation (log(x + 1)). The means equate to 50 s (water) and 8 s (morphine).

Contact with the sides of the tank in the second period was significantly affected by first treatment, with less contact recorded when the first treatment was morphine (F_1, 88_ = 43.99, *p* < 0.0001; Figure 3). Contact with the sides of the tank was not significantly affected by second treatment (F_3, 88_ = 0.861, *p* = 0.46) and there was no interaction between first and second treatments (F_3, 88_ = 1.82, *p* = 0.15).

**Figure 4:**
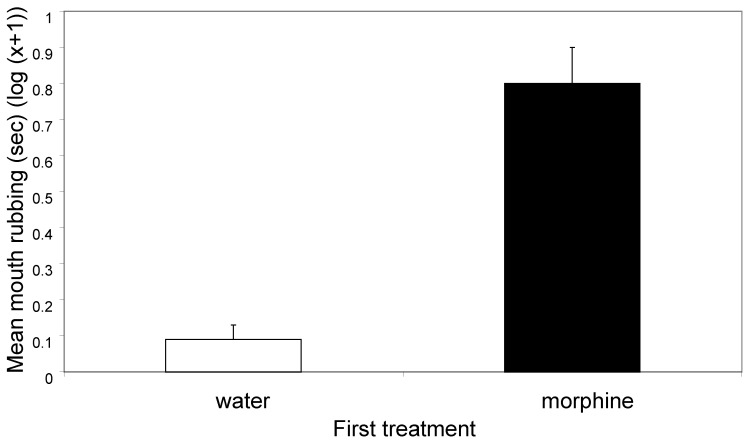
The mean (±SE) time (s) spent rubbing and picking the mouthparts with the chelipeds in the second observation (log(x + 1)). The means equate to 1.2 s (water) and 6.5 s (morphine).

There was a significant effect of first treatment on the amount of mouth rubbing during the second observation, with those crabs that received morphine showing more rubbing than those that received distilled water (F_1, 88_ = 50.25, *p* < 0.0001; Figure 4). There was no effect of second treatment on the amount of mouth rubbing (F_3, 88_ = 1.74, *p* = 0.165), and there was no interaction between first and second treatments (F_3, 88_ = 1.53, *p* = 0.212).

There were only six cases of crabs showing the defensive response during the second period, and this sample was too small for any meaningful analysis.

The occurrence of rubbing the injected leg varied significantly between concentrations of acid (0/24 for distilled water, 10/24 for 0.25% acetic acid, 7/24 for 0.5% acetic acid, 8/24 for 1.0% acetic acid; χ^2^_3_ = 12.28, *p* = 0.0065), but rubbing was not influenced by the first treatments of morphine or distilled water (15/48 versus 10/48; χ^2^_1_ = 0.35, *p* = 0.24). Vertical extension of the injected leg during the second observation also varied significantly with concentration of acid (0/24 for distilled water; 5/24 for 0.25% acetic acid, 9/24 for 0.5% acetic acid, 10/24 for 1.0% acetic acid; χ^2^_3_ = 13.78, *p* = 0.0032), but was not affected by the first treatment of distilled water or morphine (12/24 versus 12/24; χ^2^_1_ = 0.000, *p* = 1.0). The incidence of autotomy also varied significantly between concentrations of acid (0/24 for distilled water, 2/24 for 0.25% acetic acid, 20/24 for 0.5% acetic acid, 20/24 for 1.0% acetic acid; χ^2^_3_ = 61.46, *p* = 0.0079), but was not influenced by the first treatment of morphine or distilled water (23/48 versus 19/48; χ^2^_1_ = 0.677, *p* = 0.41). Sometimes the autotomy occurred before the animal could be returned to the observation tank (fast), but for others it occurred after the animal was returned to the observation tank (slow). There was no effect of morphine treatment on the speed of autotomy (16 fast; 7 slow for water treatment, 10 fast and 9 slow for morphine treatment χ^2^_1_ = 0.65, *p* = 0.42).

## 4. Discussion

### 4.1. Effects of Morphine on Behaviour

The first treatment with morphine or distilled water had marked effects on the behaviour of the animals in the first and the second observation periods. Morphine treatment caused animals to spend less time in contact with the walls of the tank in period one (Figure 1) and in period two (Figure 3). Further, morphine elicited more rubbing and picking at the mouthparts in period one (Figure 2) and in period two (Figure 4). The two observation periods were 5 min each and about 1 min was required for the injection into a leg prior to the second periods, so these observations all took place within 11 min of the morphine or control injections.

Effects on the behaviour of decapods following morphine administration have been noted in previous studies. Shore crabs, *C. maenas*, injected with morphine reduced the use of dark shelters to escape from bright light [16] for about 20 min. This fits with the present observation of reduced seeking of shelter as seen by less contact with the walls of the tank. Reduced seeking of shelter has been explained as a reduced ability to walk [16] but it could be a reduced motivation to seek shelter. Morphine injection reduced the escape response to a moving shadow in *C. granulatus*, which might have been due to a general reduction in perception or ability to move [15]. Further, the picking and rubbing of mouth parts following morphine injection was like observations on crayfish, *Orconectes rusticus*, which showed stereotypic behaviour, including grooming, tail flipping, mouthpart movements, repetitive actions, and mild tremor [20].

In the present study, morphine caused more animals to show the defensive display in period one, but not in period two, when very few animals showed these displays. Morphine seems to reduce shelter use and replace it with defensive displays. However, the increase in protective displays was short-lived. This could be because the effects of morphine declined rapidly, or it could be due to a rapid habituation to the novel environment. Regardless, we see that morphine has various effects on the behaviour of decapods, but it is not clear how morphine works on the nervous system of decapods to cause those effects [21].

### 4.2. Effects of Acetic Acid

Injection of acetic acid, of any dilution, into a joint of a hind leg caused marked changes in behaviour. The crabs were more likely to rub the injected leg with other legs and to hold the injected leg vertically. Because these activities were not seen when distilled water was injected, they cannot be caused by the minor tissue damage from the injection; rather, the acetic acid caused the effect. Acetic acid has long been used in studies on possible pain in vertebrates [3] and crustaceans [5,7], and behaviour directed at the site of noxious stimulation has been taken to be consistent with the idea of pain experience [22,23,24]. Those previous studies on crustaceans, however, involved external application by brushing acetic acid solution onto an antenna, an eye or mouth parts. The present study involved injection of acetic acid and thus was more like a study on trout that showed elevated rubbing and abnormal activities when the lip of the fish was injected [3]. It was also like a study on the shore crab, *H. sanguineus*, in which claw-bearing limbs were injected with formalin, and shaking, rubbing and unusual postures of that limb occurred [9]. These studies have demonstrated that both acetic acid and formalin causes decapods to show distinct activities that are consistent with the idea of pain, and similar effects have been noted with caustic soda brushed onto an eye in glass prawns [8].

Acetic acid injection also caused animals to autotomize the injected leg, and this occurred primarily with the higher concentrations of acetic acid. Sometimes this autotomy occurred almost immediately following acetic acid injection, i.e., before observations could commence for that period, and this obviously precluded holding the leg vertically and/or rubbing the leg. However, when autotomy occurred after the animal was placed back into the observation chamber, rubbing and holding the leg vertically often preceded autotomy. Rubbing and holding the leg vertically could also occur without autotomy and this was particularly the case with the lowest concentration of acetic acid. It seems that low concentrations of acetic acid induced leg-directed activities, but a stronger solution was usually required for autotomy. In the present study, animals were observed to rub the site of autotomy for prolonged periods (60 s), which is consistent with the idea of pain. Autotomy was also seen in *H. sanguineus* when the clawed appendage was injected with formalin [9], and, like the present study, that was often preceded by shaking and rubbing the appendage. Autotomy is a well-known response of decapods to noxious stimuli to an appendage and occurs in response to being held [25], heat [26], electric shock [16], a small cut at a joint [27], injection of dopamine [28], as well injection with formalin [9] and injection with acetic acid reported here. There has been speculation that autotomy in arthropods is mediated, at least in some cases, by a pain-like state [10,22,29]. Spiders, *Argiope* spp., for example, will autotomize a leg when injected with chemicals known to cause pain in humans but not others that do not cause pain in humans [30]. On the other hand, there is no evidence to suggest that autotomy due to being held by an appendage is mediated by pain; rather, it seems to be a decision to pay a cost to escape from a potential predator. The present results, however, are suggestive of pain-like mediation because autotomy is often preceded by rubbing and holding in an unusual posture, which are activities that fit a criterion of pain.

Autotomy in decapods occurs at the joint between the coxa and the basis (basi-ishiopodite). It is caused by contraction of muscles normally used in locomotion and fractures a specific plane that heals rapidly [31,32]. The muscles are activated by motor neurons, and thus, autotomy seems to follow central decision making [32]. It is thought that autotomy following dopamine injection is due to the dopamine acting at the site normally controlled by motor neurons [28]. Autotomy appears to vary with other motivations and the decision to autotomize may include information about costs and benefits of losing the limb [33]. Thus, it should be amenable to experiments that examine trade-offs, a key criterion of pain. Because there is a cost to autotomy, coupled with the cost of subsequent regeneration of the appendage [25], then it would be expected to be induced following a decision-making process that maximizes the fitness pay-off. This could be a better outcome than that achieved by a standard reflex response [34].

### 4.3. Effects of Morphine on Responses to Acetic Acid

Although morphine influenced the behaviour of crabs, there is no indication that it affected responses to the injection of acetic acid. That is, it did not reduce rubbing of the injected appendage or holding the appendage in a vertical position. These activities directed at the site of a noxious stimulus have the appearance of being consistent with pain, but there is no analgesic effect of morphine. Further, there was no effect of morphine on the probability of autotomy or the speed of autotomy. This might be due to an absence of any pain-like feeling, or it could be that the physiology of crabs is very different from that of vertebrates with respect to responses to morphine. Morphine has analgesic effects in vertebrates [1,2,3], but the evidence for such effects in crustaceans is weak or lacking [35]. However, local anaesthetics, such as benzocaine, are effective in reducing responses to noxious stimuli in decapods [5,8]. Benzocaine blocks the transmission of action potentials along axons, whereas opiates block transmission across synapses by inhibiting specific neurotransmitters [35].

Studies on mantis shrimps, *S. mantis*, and the crab, *C. granulatus*, have shown reduced responses to electric shock following morphine injection [13,14], and this was reversed by the opioid antagonist, naloxone [14]. However, morphine also reduced the responsiveness to visual stimuli [15] and thus there was the possibility that morphine had effects on locomotor or perceptual abilities. One experiment on shore crabs that had the expectation of greater movement into a dark chamber if there was an analgesic effect of morphine found no analgesic effect. Rather, morphine had general effects on behaviour that reduced mobility, and perhaps the effects of visual stimuli [16]. We have shown in the present study that morphine can alter behaviour, but there is no indication that it provided analgesia, because of the lack of effect on any behaviour directed to or involving the injected leg.

## 5. Conclusions

Crabs show behavioural changes to both morphine and acetic acid, but these are independent of each other. That is, morphine does not influence the responses to acetic acid and acetic acid does not modify the responses to morphine. However, the responses to acetic acid are directed at the specific limb that was injected, and directed behaviour has been accepted as a criterion for pain [22,23,24,36]. This measure is used extensively as part of the formalin test in pharmaceutical investigations [37] in which the level of activities directed at the site of injection in a leg is used as a measure of pain in rodents. Further, autotomy of the limb injected with a noxious chemical, but not with distilled water, has been suggested as an indicator of pain [10,22,29]. Thus, our present findings are consistent with the idea of pain in decapods. However, pain is impossible to prove in non-verbal species and we do not claim to demonstrate pain with these findings. Nevertheless, there is a body of studies on potential pain in decapods that test various criteria for pain, and these too have fulfilled those criteria and thus are also consistent with the idea of pain. For example, we see swift avoidance learning [38,39,40]. There are trade-offs between avoidance of a noxious stimulus and other motivational requirements, such as hermit crabs, *Pagurus bernhardus*, accepting electric shock to keep high-quality shells [41,42], or to avoid predators [43]. Further, shore crabs accept electric shocks to avoid bright but not dull light [39]. Following electric shocks, decapods show signs of anxiety [40,44,45] and show physiological stress responses after electric shocks [44,45,46] or injury [27]. Hermit crabs that are shocked within their shells show long-term reduction in their motivation to retain that shell (24 h) [47]. It is the accumulation of studies that are consistent with the idea of pain in decapods that has led to an increasing acceptance of the possibility of pain in this taxon [24]. This increasing acceptance is exemplified by the policy statement by the British Veterinary Association (2020) [48] and the recent inclusion of decapods in the Animal Welfare (Sentience) Act (2022) of the United Kingdom [49]. Although this act recognizes decapods as sentient, it has not yet resulted in changes in legal requirements to improve their welfare in scientific research or in aquaculture, fishing, transport or use in food preparation within the United Kingdom. Finally, we recognize that decapods are a diverse group and that indicators, suggestive of pain, have not been investigated in all of the major groups [24,50].

## Data Availability

Data are contained within the article.
